# Prognostic role of pretreatment [^18^F]FDG PET/CT in patients affected by adrenocortical carcinoma and treated with chemotherapy

**DOI:** 10.1007/s00259-026-07794-6

**Published:** 2026-03-06

**Authors:** Francesco Dondi, Davide Lorenzo Bettini, Deborah Cosentini, Roberta Ambrosini, Sara Rodella, Benedetta Trevisan, Andrea Abate, Mariangela Tamburello, Valentina Cremaschi, Giovanni Casole, Guido Alberto Massimo Tiberio, Sandra Sigala, Salvatore Grisanti, Francesco Bertagna, Alfredo Berruti, Marta Laganà

**Affiliations:** 1 Adrenal Cancer Unit ASST Spedali Civili, Piazzale Spedali Civili 1, Brescia, 25123 Italy; 2 Nuclear Medicine, Università Degli Studi di Brescia and ASST Spedali Civili di Brescia, Brescia, 25123 Italy; 3 Medical Oncology, Department of Medical and Surgical Specialties, Radiological Sciences, and Public Health, University of Brescia, ASST Spedali Civili, Brescia, 25123 Italy; 4 Radiology, ASST Spedali Civili, Piazzale Spedali Civili 1, Brescia, 25123 Italy; 5 Department of Molecular & Translational Medicine, Section of Pharmacology, University of Brescia, Brescia, 25123 Italy; 6 Surgery, Department of Clinical and Experimental Sciences, University of Brescia, ASST-Spedali Civili, Brescia, 25123 Italy

**Keywords:** [^18^F]FDG, ACC, Adrenocortical carcinoma, PET, Positron emission tomography, Prognostic factor

## Abstract

**Purpose:**

[^18^F]-fluorodesoxyglucose ([^18^F]FDG) positron emission tomography/computed tomography (PET/CT) is used for diagnosis and staging of adrenocortical cancer (ACC), its prognostic role needs to be explored. This study aimed to investigate the prognostic role of pre-EDP-M [^18^F]FDG PET/CT parameters in ACC patients treated with first line chemotherapy (ChT) in term of progression free survival (PFS) and overall survival (OS).

**Method:**

this retrospective, monocentric study included metastatic ACC patients treated with EDP-M after [^18^F]FDG PET/CT staging. Clinico-pathological features and PET/CT semiquantitative parameters such as standardized uptake value (SUVmax), metabolic tumor volume (MTV), total lesion glycolysis (TLG), SUVmax/liver ratio (SL) and SUVmax/blood-pool ratio (SBP) were collected.

**Results:**

forty-one ACC patients were included. Median PFS was 9.3 months and median OS was 14.3 months. PET/CT parameters did not correlate with the response to ChT, while they were affordable prognosticators. Lower MTV (HR 0.36, CI: 0.17–0.74; *p* < 0.01), lower TLG (HR 0.31, CI: 0.15–0.64; *p* < 0.01), lower tumour burden (HR 0.45, CI: 0.23–0.94; *p* = 0.03), attained the statistical significance in univariable analysis in terms of longer PFS, lower TLG (HR 0.33, CI: 0.15–0.71; *p* < 0.01) confirmed its independent role in multivariable analysis. At univariable analysis lower MTV (HR 0.36, CI: 0.16–0.82; *p* = 0.01) correlated with longer OS. Lower TLG confirmed its role in the multivariate analysis (HR 0.28, CI 0.11–0.69; *p* < 0.01).

**Conclusion:**

pretreatment TLG was reported as independent prognostic tools for ACC patients treated with EDP-M. No correlations between PET/CT parameters and response to ChT were reported.

## Introduction

Adrenocortical carcinoma (ACC) is a rare endocrine malignancy with an estimated incidence of 0.7-2 new cases per million people per year [1]. Surgery is the gold standard for localized ACC and adjuvant therapy is indicated for cases with higher risk of recurrence basing on Ki67 expression, age, cortisol hypersecretion or stage, which define the prognostic GRAS score [2–3]. Mitotane combined with polychemotherapy (EDP-M) is the current first line treatment for metastatic ACC [4–5]. This treatment strategy has a limited efficacy, although a proportion of patients can obtain a long term disease control [6]. Identifying prognostic markers and predictive parameters of efficacy is therefore crucial. Proliferative activity measurement in ACC patients before initiating EDP-M could serve as a valuable prognostic factor and predictive parameter of treatment efficacy. Proliferative status can be assessed by evaluating the immunohistochemical expression of Ki67, but it is an invasive procedure that is not commonly performed. The use of a non-invasive diagnostic tool to evaluate tumor proliferation and aggressiveness before systemic treatment is therefore of great importance. Several studies have shown a positive correlation between [^18^F]-fluorodesoxyglucose ([^18^F]FDG) uptake (measured as standardized uptake value [SUV]) and tissue Ki-67 expression in many malignancies [7]. Higher Ki-67 expression often corresponds to higher [^18^F]FDG uptake, reflecting a more metabolically active, rapidly proliferating tumors [8]. Ki-67 is routinely available and reproducible at diagnosis. However, in metastatic ACC, repeat biopsy to reassess Ki-67 during progression is not always feasible. While limited data exist on its temporal variation, some studies suggest that Ki-67 may increase at relapse, reflecting tumor dedifferentiation or decrease after response to cytoreductive chemotherapy (ChT) [6].

Current international guidelines suggest [^18^F]FDG positron emission tomography/computed tomography (PET/CT) as complementary tool in addition to conventional imaging techniques to define extension of the adrenal tumour and evidence for metastases, especially bone. [^18^F]FDG PET/CT uptake has been shown to be prognostic in different neoplasms [9–10].

The prognostic role of [^18^F]FDG PET uptake in patients with ACC has been assessed in a few studies involving newly diagnosed patients, but the results have been conflicting. In two studies, a higher SUVmax, body metabolic tumor volume, and total lesion glycolysis (TLG) were associated with shorter survival [11–12]. However in 2 more recent studies, quantitatively derived PET and CT parameters, failed to be significantly associated with OS [13–14].

To our knowledge there are no studies that have assessed the role of [^18^F]FDG PET/CT as a prognostic and predictive parameter of efficacy in metastatic patients treated with ChT. In this paper we report the results of a retrospective study conducted at the Adrenal Unit of ASST-Spedali Civili in which we evaluated the prognostic and predictive role of [^18^F]FDG PET/CT in a consecutive series of metastatic ACC patients uniformly treated with the EDP-M scheme. In particular, two major points were analyzed in the paper: the predictive ability of pre-treatment [18F]FDG PET/CT with respect to the response to ChT and the prognostic role of pre-treatment PET on the long-term outcome of patients treated with ChT.

## Patients and methods

### Patients selection

**Fig. 1:**
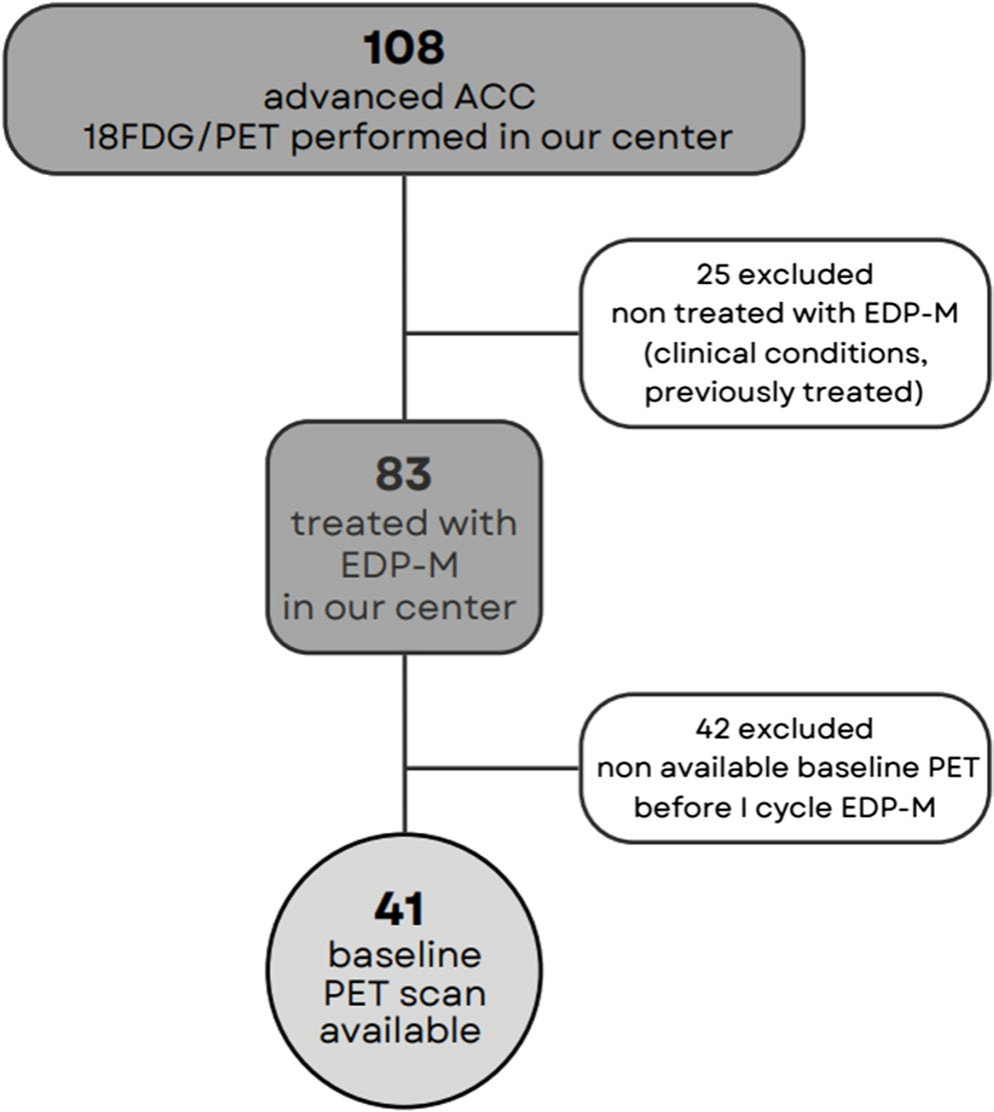
Consort diagram

Our database was retrospectively screened to retrieve advanced patients submitted to EDP-M who had performed [^18^F]FDG PET/CT for the baseline staging of ACC from January 2014 to December 2023. Among 83 metastatic ACC patients treated with EDP-M in our centre who underwent at least one [^18^F]FDG PET/CT scan, 41 had this baseline-scan available, performed before starting ChT and were included in the study (Fig. 1).

Clinicopathological information about gender, age, functional status of ACC, European Network for the Study of Adrenal Tumours (ENSAT) stage, tumor burden, Ki67 value, type of ChT were collected. Informed consent was obtained from all individual participants included in the study.

### [^18^F]FDG PET/CT acquisition and interpretation

Before PET/CT acquisition patients fasted for at least 6 h and they had a glucose blood level below 150 mg/dL at the time of injection; 3.5–4.5 MBq/kg of [^18^F]FDG were intravenously injected to the patients and before images acquisition they were instructed to void. No contrast agent, intestinal preparation with purge or enteric contrast were used. Images were acquired 60 min after radiotracer injection, from the skull base to the midthigh on a Discovery ST or Discovery 690 PET/CT tomograph (General Electric Company, GE, Milwaukee, Wisconsin) in the same department with standard parameters (CT: 80 mA, 120 kV; PET: 2.5–4 min per bed position, PET step of 15 cm). Images were reconstructed with a 256 × 256 matrix and a 60 cm field of view. On Discovery 690 tomograph time of flight (TOF) and point spread function (PSF) algorithm were used for the reconstruction of images, with filter cut-off 5 mm, 18 subsets and 3 iterations. For Discovery ST tomograph, an ordered subset expectation maximization (OSEM) algorithm with filter cut-off 5 mm, 21 subsets and 2 iterations was used.

PET/CT images were visually and semiquantitatively analyzed by two experienced nuclear medicine physicians by consensus. Every focal tracer uptake deviating from physiological distribution and from the background was regarded as suggestive of disease localization. The semiquantitative analysis of images was performed by measuring SUVmax of the most hypermetabolic lesion. Furthermore, the SUVmax of the liver was calculated at the VIII hepatic segment from transaxial PET images using a spheric volume of interest (VOI) with 1 cm diameter. The SUVmax of the blood-pool was calculated at the aortic arch by using transaxial PET images with a similar VOI, paying attention to not involve the vessel’s walls. These values were used to calculate a ratio between SUVmax of the lesion with the highest uptake and these two parameters (SL and SBP, respectively).

An SUV-based automated contouring program (Advantage Workstation 4.6, GE HealthCare) was used to measure metabolic tumor volume (MTV) from attenuation corrected [^18^F]FDG PET/CT images, using an isocontouring threshold method based on 41% of the SUVmax, as recommended by the European Association of Nuclear Medicine guideline [15]. Furthermore, total lesion glycolysis (TLG) was calculated as the sum of the product of the MTV of each lesion and its SUVmean.

### Statistical analysis

All statistical analyses were performed using SPSS v29.0.1.0 for Windows (SPSS Inc, Chicago, USA). The descriptive analysis of categorical variables comprised the calculation of simple and relative frequencies. The numeric variables were described as median and minimum and maximum (range).

Overall Survival (OS) was defined as the time in months from the date of the initiation of ChT to the date of death for any cause or to the date of last documented follow-up. Progression Free Survival (PFS) was calculated as the time in months between the initiation of ChT and the date of first documented disease progression, based on radiological imaging (x-ray, CT, magnetic resonance and PET/CT).

Logistic regression analysis was used to evaluate a correlation between PET parameters and the response to ChT, considering stable and responding patients vs. progressing disease defined with the combination of PET/CT and conventional imaging data.

Kaplan-Meier analysis was used to draw survival curves which were compared using the log-rank test. A p-value < 0.05 was considered as statistically significant. In order to perform such analyses, continuous quantitative parameters were dichotomized based on their median value. This decision has been made given the small sample of our cohort, retaining that a median-based dichotomization could avoid model overfitting. Furthermore, Cox regression model was applied to identify independent prognostic factors between clinicopathological and PET/CT features. In particular, these features were retained for multivariable analysis if they had a significant prognostic value at univariable analysis (p-value < 0.05) or they were near the limit of statistical significancy (p-value < 0.10). Estimates of the predictive effect for OS and PFS were expressed as hazard ratios (HRs) in univariable and multivariable Cox regression analyses with a 95% confidence interval (CI).

## Results

### ACC patients characteristics and [^18^F]FDG PET/CT scan semiquantitative parameters

All the patients included in the study were metastastic at the moment of [18 F]FDG PET/CT performed before ChT start. Among 41 included patients, the median age at time of baseline-PET was 53 years (range 24–77) and 27 (66%) patients were female. Thirty-one patients (75%) were not metastastic at ACC diagnosis while 6 (14%) presented a mEnsat stage IVa, 2 (5%) stage IVb and 2 (5%) stage IVc. ACC was diagnosed due to hormone hypersecretion symptoms in 15 (37%) patients and due to tumor mass effect in 12 (30%), while 3 (7%) patients presented both hormone hypersecretion and mass effect symptoms; in 11 (26%) patients the diagnosis was incidental. Among 28 patients for whom the plasma cortisol value was available at the time of the PET pre-ChT, 14 patients (50%) had cortisol hypersecretion. At diagnosis, median Ki-67 value was 20% (range 3–65), 5 (6%) patients had a lower value less than 10% (ki67 value was available in 39 patients). At ChT start 18 patients (44%) presented 1 metastatic site while 23 (56%) had the involvement of 2 or more organs other than the adrenal gland. These patients were defined as having high tumour burden (Table 1).

**Table 1:** Patients characteristics

*N* = 41	
Clinicopathological charachteristics	*N* (%) or Median (range)
Age at baseline-PET	53 (24–76)
Sex	
− Male − Female	14 (34) 27 (66)
Diagnosis	
− Hormonal related symptoms − Mass related symptoms − Both hormonal and mass related symptoms − Incidentaloma	15 (37) 12 (30) 3 (7) 11 (26)
Cortisol secretion at PET pre CHT*	
− Yes − No	14 (50) 14 (50)
Serum cortisol levels at PET pre CHT	7.00 (1.00–29.00)
mENSAT stage at diagnosis	
− 1 − 2 − 3 − 4a − 4b − 4c	2 (5) 17 (41) 12 (29) 6 (14) 2 (5) 2 (5)
Ki67 at ACC diagnosis**	
− < 20 − ≥ 20	18 (46) 21 (54)
Surgery on primary ACC	30 (73)
Adjuvant mitotane treatment	20 (49)
Tumor burden at the start of CHT and PET evaluation	
− 1 site − 2 sites − 3 sites − 4 sites − 5 sites	18 (44) 11 (27) 6 (15) 5 (12) 1 (2)
Disease site	
− Liver − Lung − Adrenal lodge − Peritoneum − Lymphnodes − Bone − Kidney	22 (54) 22 (54) 13 (32) 11 (27) 5 (12) 4 (10) 2 (5)

*Data available for 28 patients

**Data available for 39 patients

*N* number, *ENSAT* European Network for the Study of Adrenal Tumours, *ACC* adrenocortical carcinoma, *PFS* progression free survival, *OS* Overall survival, *CHT* chemotherapy, *EDP* etoposide, doxorubicine and cisplaTin.

The median time interval between [^18^F]FDG PET/CT and first line ChT start was 1 day [1–30]. Most of the patients (34/41, 82.9%) performed PET/CT imaging on Discovery 690 tomograph.

As for the [^18^F]FDG PET/TC semiquantitative parameters, median SUVmax was 12.2 (range 3.3–42.2), median MTV was 41.1 (range 2.6-2011.8), median TLG was 239.9 (range 11.6–8800), median SBP was 5.9 (range 2.1–27.4) and median SL was 3.9 (range 1.1–16.3). No significant differences were reported for such parameters between responders patients and non responders subjects (Table 2). Additionally, PET/CT semiquantitative parameters at chi-square analysis showed a statistically significant correlation between higher TLG and tumor burden (p-value 0.03) (Table 3). 

**Table 2:** Summary of PET/CT semiquantitative parameters (median (range))

Parameter	All patients (*N*: 41)	Non responders (*N*: 18)	Responders (*N*: 23)	*p* value
SUVmax	12.2 (3.3–42.2)	12.7 (3.3–31.3)	11.6 (4.8–42.2)	0.475
MTV	41.1 (2.6–2011.8)	42.1 (2.6–374.7)	41.1 (4.3–2011.8)	0.352
TLG	239.9 (11.6–8800.0)	213.0 (11.6–2662.7)	322.9 (13.7–8800.0)	0.263
SL	3.9 (1.1–16.3)	4.9 (1.1–8.4)	3.7 (1.7–16.3)	0.362
SBP	5.9 (2.1–27.4)	5.9 (2.3–11.8)	5.6 (2.1–27.4)	0.347

Non responders: progression of disease burden at imaging at the end of CHT.

Responders: stability or reduction of disease burden at the end of CHT.

*N* number, *SUVmax* maximum standardized uptake value, *MTV* metabolic tumour volume, *TLG* total lesion glycolysis, *SL* SUVmax/Liver ratio, *SBP* SUVmax/blood-pool ratio.

**Table 3:** Correlation between [^18^F]FDG PET/CT semiquantitative parameters and clinicopathological features (R value)

Variable	SUVmax	SL	SBP	MTV	TLG
Cortisol	0.154	0.165	0.064	0.239	0.265
Ki67%	0.008	0.0003	-0.011	0.031	0.089
Tumor burden	0.083	0.108	0.127	0.038*	0.458**

* p value 0.012

** p value 0.002

*SUVmax* maximum standardized uptake value, *MTV* metabolic tumour volume, *TLG* total lesion glycolysis, *SL* SUVmax/Liver ratio, *SBP* SUVmax/blood-pool ratio.

### [^18^F]FDG PET/CT pre-chemotherapy scan parameters correlation with response

All patients started ChT according to the EDP-M regimen (median number of cycles 5, range 1–10) after [^18^F]FDG PET/CT scan [16]. Thirty-one (76%) patients presented mitotane level in range during the ChT treatment. The best response was complete response in 3 subjects (7%), partial response in 13 patients (32%), stable disease in 7 (17%), progression in 18 (44%).

Logistic regression analysis revealed that none of the assessed PET parameters demonstrated a statistically significant correlation with the response to ChT, considering stable and responding patients vs. progressing disease. In particular, TLG had an odds ratio (OR) of 2.365 (95% confidence interval [CI] 0.200-27.929) with a p value of 0.494, MTV an OR of 1.953 (95% CI 0.452–8.442) with a p value of 0.370 and SUVmax an OR of 0.118 (95% CI 0.009–1.628) with a p value of 0.111.

### Prognostic role of [18 F]FDG PET/CT pre-chemotherapy scan parameters

The median follow-up of this patient series was 42 months (range 8-161), during which 34 (83%) patients experienced disease progression and 27 (66%) died. The median PFS after ChT start was 9.3 months (range 1.8–64.9) and the median OS was 14.3 months (range 1.5–79.6).

**Fig. 2:**
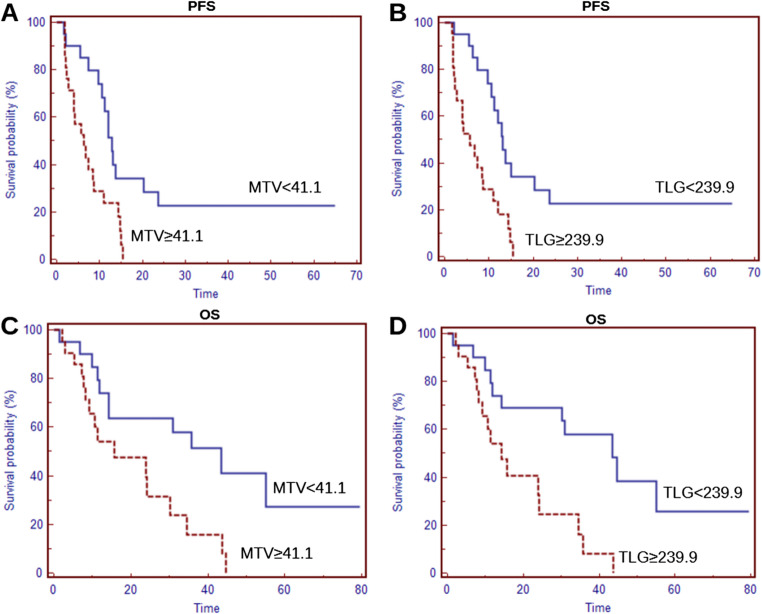
Survival curve analyses for PFS and MTV (p 0.004)(**A**), PFS and TLG (*p* < 0.001)(**B**), OS and MTV (p 0.011)(**C**) and OS and TLG (p 0.003)(**D**)

Lower MTV (HR 0.36, CI: 0.17–0.74; *p* < 0.01), lower TLG (HR 0.31, CI: 0.15–0.64; *p* < 0.01), lower tumour burden (HR 0.45, CI: 0.23–0.94; *p* = 0.03), were significantly associated with a lower risk of disease progression. Since the value of MTV is included in the calculation of TLG, subsequent multivariable analysis was performed including only the latter. Lower TLG (HR 0.33, CI: 0.15–0.71; *p* = 0.005) confirmed to be an independent parameter associated with PFS at multivariable analysis (Fig. 2).

As regards as OS, univariable analysis revealed that lower MTV (HR 0.36, CI: 0.16–0.82; *p* = 0.01) and lower TLG (HR 0.30, CI: 0.12–0.69; *p* < 0.01) correlated with lower risk of death while lower tumour burden was near the statistical significance value (HR 0.48, CI: 0.21–1.07; *p* = 0.07). Lower TLG did achieve statistical significance as an independent prognostic factor in terms of OS in the multivariable analysis (HR 0.28, CI 0.11–0.69; *p* < 0.01) (Table 4).

**Table 4:** Univariable and multivariable analysis with clinical-pathological and [18 F]FDG PET/CT semiquantitative parameters for PFS and OS

	Univariate analysis		Multivariate analysis	
	*p*-value	HR (95% CI)	*p*-value	HR (95% CI)
PFS				
SUVmax lower than median	0.38	0.74 (0.37–1.45)	–	–
MTV lower than median	0.006	0.36 (0.17–0.74)	–	–
TLG lower than median	0.002	0.31 (0.15–0.64)	0.005	0.33 (0.15–0.71)
SL lower than median	0.15	0.60 (0.30–1.21)	–	–
SBP lower than median	0.19	0.62 (0.30–1.26)	–	–
No cortisol secretion	0.27	1.60 (0.69–3.72)	–	–
Age at diagnosis lower than 50y	0.54	0.81 (0.41–1.59)	–	–
Tumour burden < 2	0.03	0.45 (0.23–0.94)	0.107	0.54 (0.26–1.13)
OS				
SUVmax lower than median	0.64	0.83 (0.39–1.78)	–	–
MTV lower than median	0.01	0.36 (0.16–0.82)	–	–
TLG lower than median	0.005	0.30 (0.12–0.69)	0.006	0.28 (0.11–0.69)
SL lower than median	0.31	0.67 (0.30–1.47)	–	–
SBP lower than median	0.75	0.88 (0.40–1.92)	–	–
No cortisol secretion	0.40	0.65 (0.24–1.75)	–	–
Age at diagnosis lower than 50y	0.79	0.90 (0.42–1.93)	–	–
Tumour burden < 2	0.07	0.48 (0.21–1.07)	0.21	0.59 (0.25–1.35)

*PFS* progression free survival, *OS* overall survival, *CHT* chemotherapy, *SUVmax* maximum standardized uptake value, *MTV* metabolic tumour volume, *TLG* total lesion glycolysis, *SL* SUVmax/Liver ratio, *SBP* SUVmax/blood-pool ratio.

## Discussion

**Fig. 3:**
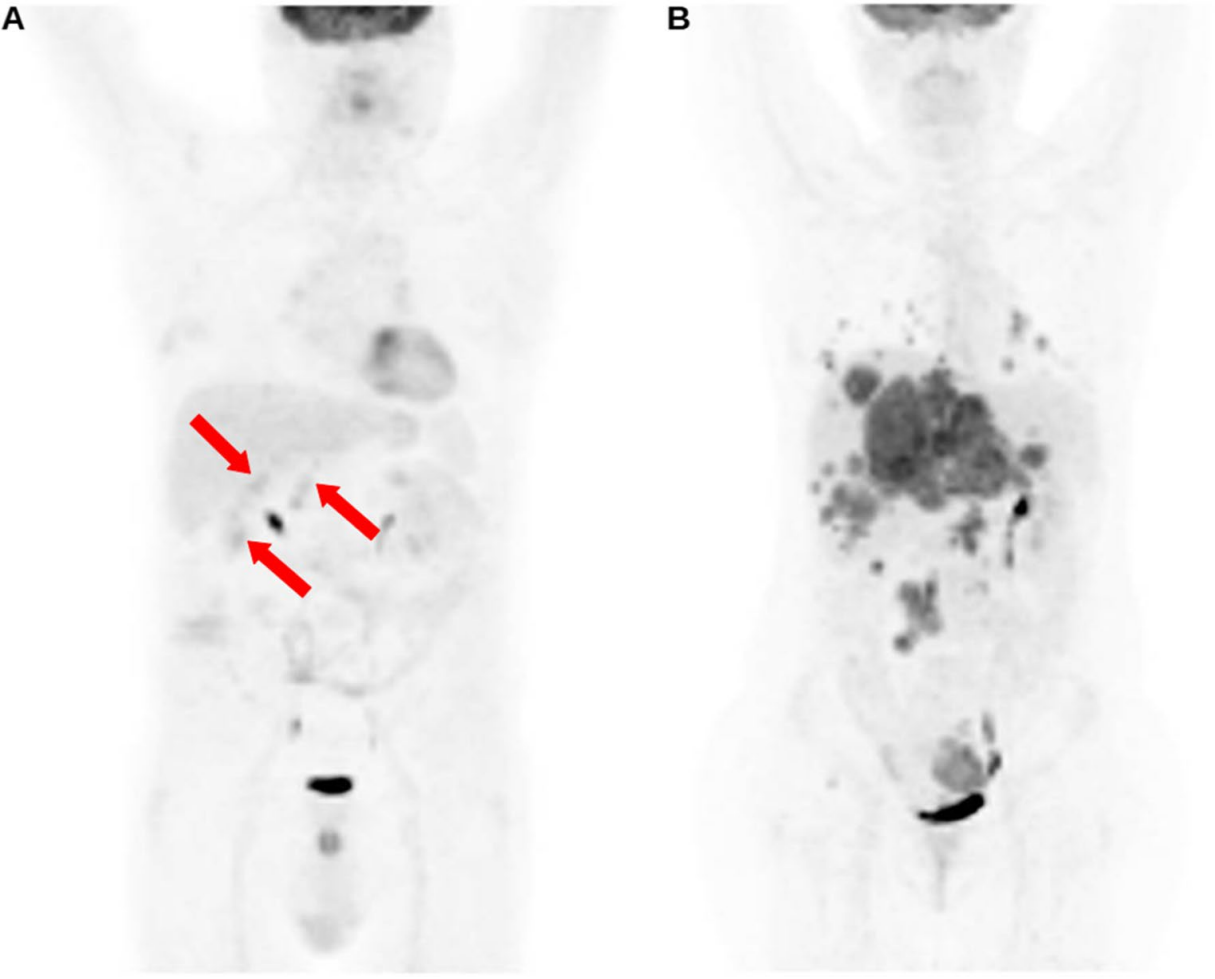
Maximum intensity projection (MIP) images of two [18 F]FDG PET/CT scans of two different patients. The first one (**A**) demonstrated the presence of foci of tracer uptake (red arrows) on primary ACC of the right adrenal gland, on paracaval lymphnodes and at the I and IV hepatic segments; the patient had an MTV of 96.0, a TLG of 239.9, a PFS of 12.1 months and an OS of 55.1 months. The second scan (**B**) demonstrated the presence of multiple foci of tracer uptake on the primary right ACC, on both lungs, at the thoracic wall, on different abdominal nodal levels, at the liver and at the bone; the patient had an MTV of 882.9, a TLG of 8186.2, a PFS of 10.9 months and an OS of 24.8 months

[^18^F]FDG PET/CT is a well established tool for the diagnosis and the assessment of recurrence of malignant adrenal tumors and its prognostic role has also been investigated in the past [17, 18–19]. In this paper we specifically focused on the predictive ability of this imaging modality to evaluate the efficacy of ChT. In this retrospective study of patients with metastatic ACC, all uniformly treated with EDP-M, baseline pre-ChT [^18^F]FDG PET/CT parameters were demonstrated as independent prognostic predictors, while they did not demonstrate a correlation with response to ChT. In particular, TLG was demonstrated to be a significant independent prognostic parameter. In addition, since the calculation of TLG comprehends MTV, also this parameters can be considered as an affordable prognostic factor. These parameters reflects the disease burden of the patients in terms of both metabolic activity and volumetric dissemination, indicating that subjects with higher amount of neoplastic mass and metabolic active disease have a worst prognosis in terms of disease progression and death. A representative case that demonstrates this correlation is shown in Fig. 3.

Our data confirm previously reported correlations between [^18^F]FDG PET/CT findings and OS [11–12, 20], with the addition that in our study the correlation was also demonstrated with PFS. This study’s results are in conflict with those of two papers that found no prognostic value of this imaging modality in ACC patients [13–14, 21]. The prognostic insights of [^18^F]FDG PET/CT in patients with ACC has been previously reported by Leboulleux et al. [20] that reported SUVmax and the volume of uptake of the tracer, a parameter similar to MTV, significantly associated with survival however without a confirmation at multivariable analysis. Similarly, Satoh et al. [12] demonstrated that subjects with higher MTV, TLG or SUVmax had a worse OS; interestingly, no significant differences in terms of OS were underlined between patients who performed ChT prior to operative intervention and those who did not perform it. More recently, Wrenn et al. [11], revealed a strong negative association between SUVmax and OS. Our results demonstrated and confirmed the prognostic role of baseline [^18^F]FDG PET/CT, in particular in the setting of patients treated with ChT. However, it should be noted that the patients included in the present study, all of whom were metastatic and treated with ChT, are different from other published case series, and this may explain the observed differences. As a matter of fact, the median SUVmax, MTV and TLG values of [18F]FDG PET/CT scans of our cohort were generally higher compared to the same values reported in literature for ACC patients, even though differences in acquisition and reconstruction parameters and extraction of volumetric metrics may explain these differences [11–12, 14, 20–21].

As expected, in our series there was a statistically significant correlation between TLG and tumor burden: these two parameters reflect the quote of neoplastic tissue, strengthening the fact that the total burden of disease, considering both metastases numbers and metabolism, predict the prognosis of ACC subjects. However TLG maintained its prognostic role after adjustment for the extension of the disease, and this evidence supports the distinct prognostic significance of the aggressiveness of the disease measured by TLG, that includes also the metabolic activity, compared to its extension.

Despite the [^18^F]FDG uptake reflects the aggressiveness of the disease, our findings did not confirm a correlation between SUVmax and Ki-67 expression. The reason for this discrepancy may be related to the fact that the majority of patients had their Ki-67 evaluation performed during the diagnosis of the disease, many months before the start of systemic treatment and before performing PET/CT evaluation.

Regarding the potential predictive role of [^18^F]FDG uptake on the efficacy of EDP-M, our data showed that none of the baseline PET parameters predicted tumor response. This underlines that the aggressiveness of the disease alone does not significantly affect sensitivity to ChT.

The present study’s strengths lie in its monocentric design and the fact that the CT and PET/CT scans were evaluated by board-certified radiologists and nuclear medicine physicians, respectively. Whereas the retrospective nature and the small sample of patients are the main limitations. In this setting, the relatively small sample size is mainly due to the rarity of metastatic ACC and additionally, because of the retrospective design, no formal sample size calculation was performed. In addition, even though our systems received regular cross-calibration, the fact that the PET/CT acquisitions were not performed with the European Association of Nuclear Medicine Research Ltd. (EARL) accreditation and therefore no harmonization between them was performed is an issue that might be kept in mind when considering the reproducibility of our findings. Despite that, several internal control measures (such as for example regular calibration with phantoms, uniformity of protocols and periodic quality controls) have been implemented to minimize this effect. Furthermore, even though the reproducibility of PET semiquantitative parameters has been underlined in the past [22], another limitation of this study is the fact that two different PET/CT tomographs have been used, although this might be the condition in different departments. In addition, another important point is that, as mentioned, the assessment of response to therapy was performed by considering both PET/CT and conventional imaging data instead of RECIST evaluation to better capture metabolic changes of the disease; this fact might introduce biases and it could be one of the reasons why no correlation has been found between baseline PET parameters and early response to treatment.

## Conclusion

[18 F]FDG PET/CT, performed prior to ChT treatment for advanced/metastatic ACC patients, provides useful predictive information on patient PFS and OS. However, the results of this imaging modality did not correlate with response to ChT. Changes in tracer uptake before and after treatment provide complementary information to the radiological response for a better definition of the efficacy of treatment in the individual patient. Validation study is needed to determine whether [18 F]FDG PET/CT is a valuable imaging test to complement routine CT scan in patients eligible for systemic antineoplastic treatment.

## Data Availability

The datasets generated during and/or analysed during the current study are available from the corresponding author on reasonable request.
